# Outer-context determinants in the sustainment phase of a reimbursement-driven implementation of evidence-based practices in children’s mental health services

**DOI:** 10.1186/s13012-021-01149-5

**Published:** 2021-08-19

**Authors:** Joyce H. L. Lui, Lauren Brookman-Frazee, Teresa Lind, Kenny Le, Scott Roesch, Gregory A. Aarons, Debbie Innes-Gomberg, Keri Pesanti, Anna S. Lau

**Affiliations:** 1 Department of Psychology, University of California, Los Angeles, Los Angeles, CA USA; 2 Department of Psychology, University of Maryland, College Park, College Park, MD USA; 3 Department of Psychiatry, University of California, San Diego, San Diego, CA USA; 4 Child and Adolescent Services Research Center, San Diego, CA USA; 5 Department of Psychology, San Diego State University, San Diego, CA USA; 6 Los Angeles County Department of Mental Health, Los Angeles, CA USA

**Keywords:** Evidence-based practice, Implementation strategy, Outer context

## Abstract

**Background:**

Although there is increasing investment to implement evidence-based practices (EBPs) in public systems across the USA, continued or sustained use of EBPs after initial implementation remains a challenge. The low integration of EBPs in routine practice severely limits their public health impact, highlighting the need to understand factors that affect the return on costly investments in EBP implementation. This study aims to (1) characterize trajectories of EBP delivery volume through a reimbursement-driven implementation and (2) examine impacts of system-level policy regulatory activity and state-level mental health services funding on the implementation reimbursement strategy.

**Methods:**

This study involved secondary data analyses. Psychotherapy administrative claims and regulatory site visit data from the Los Angeles County Department of Mental Health and California state mental health expenditures were extracted from 2010 to 2017. Multilevel regression examined EBP claims volume over time with state expenditures and regulatory compliance as predictors.

**Results:**

EBP claims volume trajectories demonstrated a rapid initial increase, followed by a period of decrease, and a small increase in the final year. State mental health expenditures increased across time reflecting increased funding availability. State mental health expenditures and system regulatory compliance were inversely related to EBP claims volume.

**Conclusions:**

The impact of reimbursement-driven EBP implementation strategy is sensitive to multiple outer-context determinants. At the system level, commitment to fidelity of implementation regulations resulted in reduced use of the reimbursement strategy. Alternative reimbursement streams not tied to EBPs coupled with an expanded array of reimbursable services also impacted the use of the reimbursement strategy to implement EBPs.

There is a strong emphasis to provide evidence-based practices (EBPs) in the USA and internationally in order to improve child and family outcomes and functioning. Despite their benefits, EBPs remain underutilized in routine mental health care [1]. Even with supports to implement EBPs, sustained use within a service setting’s normal operations after initial implementation is a challenging goal [2, 3]. In one national initiative in the USA to implement five EBPs across eight states, less than half of the initial programs showed sustained delivery of EBPs 6 years post implementation [4]. In another county-wide effort to implement EBPs for children in Los Angeles, therapists sustained delivery of any given EBP for less than 2 years [5]. The low integration of EBPs in routine practice severely limits their public health impact, highlighting the need to understand factors that affect the return on costly investments in EBP implementation.

Implementation occurs within an ecology of service delivery where many factors interact to influence implementation and sustainment. The Exploration, Preparation, Implementation, and Sustainment (EPIS) framework posits that both inner- and outer-context factors influence implementation and sustainment processes [6, 7]. Inner-context factors refer to characteristics within agencies such as organizational leadership, climate, and individual provider characteristics such as therapist perceptions of the utility of EBPs. The outer context refers to factors external to the organization/agency, including system-level leadership, policies, regulations, and legislation in the service environment; the financing of care across municipal, state, and federal levels; and inter-organizational networks [6, 7]. Research has demonstrated the importance of several outer-context determinants for the implementation and sustainment of EBPs in North America and internationally. For example, system leadership in the form of perseverance for implementing EBPs, arranging funding and contract agreements to institutionalize EBPs, and fostering ongoing collaborations between stakeholders is positively associated with sustainment of an EBP for child maltreatment in the USA [8]. Similarly, availability of continued financial support was identified as a critical determinant for sustainment of an HIV prevention program in Mexico [9]. On the other hand, insufficient coordination across service sectors [10] and decline in or insufficient funding negatively impacted sustainment of innovations in Brazil and the UK, respectively [11]. Outer-context factors have been largely understudied in the literature relative to inner-context factors [7, 12]. Systematically examining the influence of outer-context factors on sustainment outcomes can inform future efforts in promoting long-term returns on EBP initiatives.

Implementation science is increasingly focused on testing the effectiveness of implementation strategies, defined as methods and techniques to increase EBP adoption, implementation, and sustainment outcomes [13]. Most studies examined inner-context implementation that focuses on provider education and training strategies (e.g., supervision, consultation) [14–16] or improving organizational climate (e.g., leadership training) [17]. However, outer-context fiscal implementation strategies, such as allowance structures that place EBPs on a fee-for-service list for reimbursement or incentivization through enhanced reimbursement rates for EBPs [16], have not been systematically examined, despite being applied in various service systems [15]. Importantly, EPIS identifies the importance of bidirectional “bridging factors” that link outer and inner contexts (e.g., contracting arrangements between a system and agency that regulates the terms of an implementation effort) [6, 7, 18]. Lengnick-Hall et al. [18] posited that contracting arrangement is a type of bridging factor where service systems “communicate, interact, and exchange resources” with organizations within the system to influence EBP implementation, such as by specifying eligibility requirements and billing and outcome reporting processes.

Understanding the impact of fiscal implementation strategies is critical, as there is evidence that implementation initiatives may falter when there is misalignment between EBP innovation and funding mechanisms meant to pay for them [19]. In addition, little is known about how fiscal implementation strategies may be affected by outer-context determinants including the broader funding landscape and regulatory and policy oversight. Although investigators have commented on the challenging reality of implementing EBPs under constrained financial resources in community mental health [20, 21], no study has systemically examined how changes in a state’s fiscal landscape may impact a system-driven fiscal implementation strategy. Implementation of EBPs through placement on formulary lists for fee-for-service reimbursement may be particularly sensitive to the fiscal outer context. This type of fiscal implementation strategy may be potent when other funding sources are limited or unavailable. The outer context of funding for public mental health is subject to some volatility due to state and county tax revenues [22, 23], and it is possible that outer context changes may shape the outcomes of reimbursement-driven implementation.

In addition to fiscal support, the service environment is another important outer context to consider for EBP implementation and sustainment. Regulations and policies mandating or supporting the use of EBP can facilitate the uptake of EBP. For example, Oregon has legislation that mandates 75% of treatment expenditures to be on EBP delivery, which encouraged EBP implementation [24]. Furthermore, implementation oversight has been identified as a critical component to policymakers when implementing EBP initiatives, which involves developing implementation standards or regulations, creating systems to monitor compliance with implementation over time, and providing feedback to individual programs for improvement [25]. Although research has examined audit and feedback systems to improve adherence to clinical practice guidelines [26], less is known about the effects of such regulatory oversight in implementation as usual settings. Regulatory activities in the administration and oversight of a system-driven EBP implementation may likely impact the outcomes of a reimbursement implementation strategy and corresponding EBP implementation and sustainment.

## Current study and context

The current study occurred in the USA, where health care is situated in a multi-payer system, with third-party payers including governments and commercial health insurers reimbursing providers for rendering care [27]. The most common form of reimbursement is fee-for-service, where providers are reimbursed based on unit of services or procedures provided. The Los Angeles County Department of Mental Health (LACDMH), the largest county public mental health system in the USA, utilizes such a fee-for service system. In 2004, the Mental Health Services Act (MHSA) was passed in California to impose a 1% tax on individuals’ income in excess of $1 million, which would fund Prevention and Early Intervention (PEI) services, community services and supports, workforce education and training, and innovations [28]. However, California experienced a significant state budget crisis beginning in 2008 that resulted in a state budget shortfall in the billions. Usual funding streams, especially County General Funds, were significantly curtailed, threatening the imminent closure of many mental health agencies in Los Angeles county. At the same time, new funding through MHSA became available. In fiscal year 2009–2010, agencies contracted with LACDMH were given the opportunity to receive funding through the Prevention and Early Intervention (PEI) program within MHSA. Provider training for PEI services began in March 2010, and services were offered beginning July 2010. Beginning in 2014, the economy recovered, and state funding streams for previously routine sources of reimbursement were restored (e.g., Medicaid Early Periodic Screening, Diagnosis, and Treatment; Medicaid; County General Funds). The significant fluctuations in state funds allowed for the opportunity to examine how changes in fiscal funding at the state level impacted EBP implementation and sustainment.

The PEI initiative involved a fee-for-service reimbursement implementation of EBPs for children’s mental health. LACDMH offered agencies the opportunity to receive reimbursement for the delivery of approved EBPs under the PEI initiative. In this way, the county-level contractual arrangement represented a fiscal implementation strategy to promote the use of EBPs through contracts for PEI services. During the state budget shortfall, PEI funds allowed for the continuation of mental health services, although use of the funds required the delivery of approved EBPs. The eventual recovery of general funding expanded opportunities for therapists to claim for services that were not tied to delivering EBPs required under LACDMH’s PEI initiative. Further, the process of contracting for EBP delivery by LACDMH represents a bridging factor between the outer-context policy to fiscally support EBPs and the inner-context implementation of the policy through a reimbursement structure.

As the PEI initiative unfolded, state authorities promulgated regulations to further delineate the components and eligibility criteria for PEI, codifying guidelines in 2015. LACDMH conducted two rounds of site visits between 2012–2013 and 2014–2016. The first round focused on technical assistance in supporting agencies in the ramp-up of PEI services; the second round focused on monitoring and compliance with PEI implementation guidelines. The timeline of regulatory monitoring within the county service system presented a unique opportunity to also examine the impact of regulatory monitoring on the service delivery outcomes of the reimbursement-driven EBP implementation.

### The current study

**Fig. 1:**
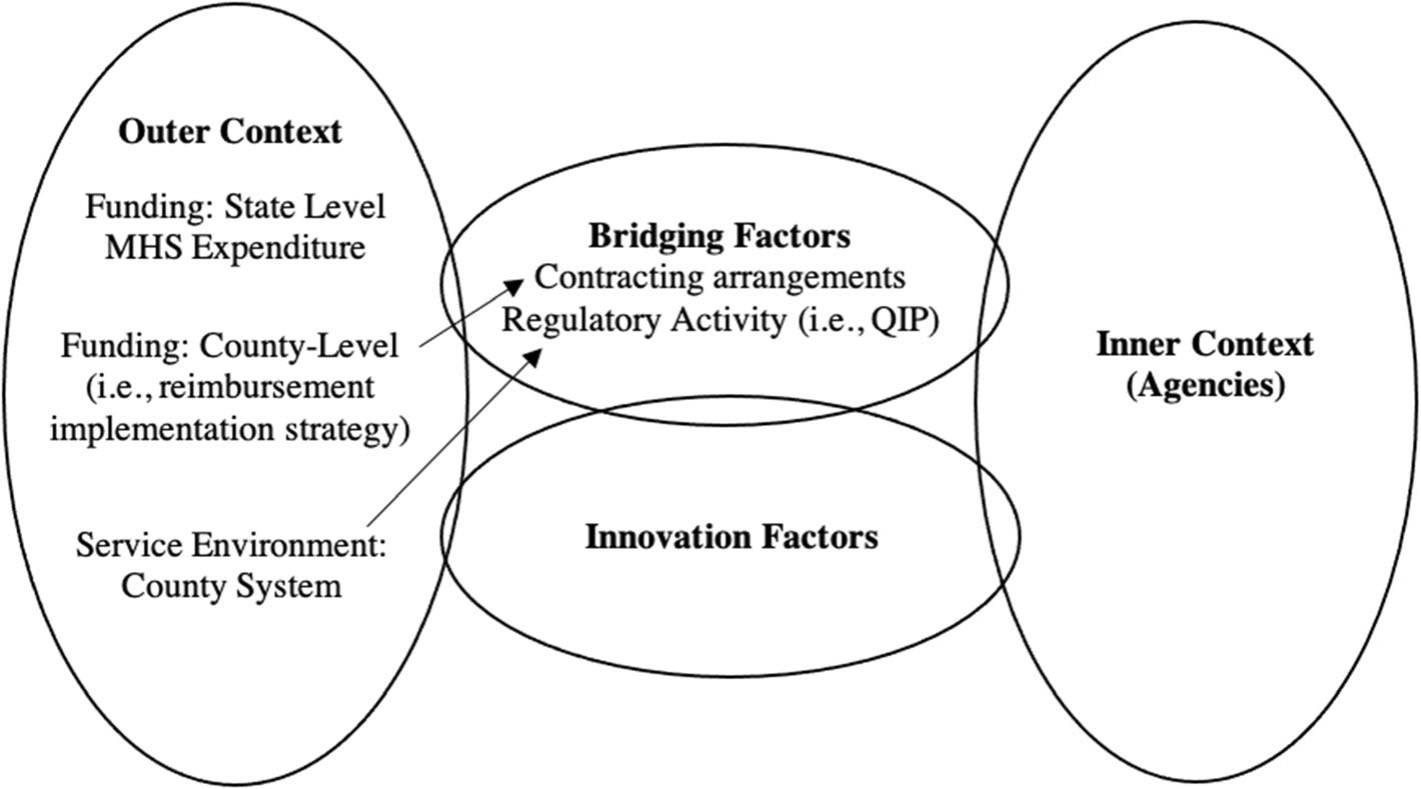
EPIS framework with study variables. Note: Adapted from Moullin et al. [7]

This study examined how changes in outer-context funding and regulatory activities in LA County, California from 2010 to 2017 impact the sustained outcomes of a reimbursement-driven EBP implementation strategy. Drawing on the EPIS model, state-level MHS expenditure and system regulatory activity are considered outer-context determinants, and the reimbursement-driven implementation strategy targets the outer context and bridges the state and county level with individual agencies (Fig. 1). This is accomplished through “bridging factors” of contracting agreements and regulatory activities that link outer context policy with directives and incentives for mental health agencies [18, 29]. Aim 1 sought to describe the volume of EBP delivery indexed by administrative claims within a reimbursement-driven implementation strategy over 8 years. The trajectory of EBP delivery volume was characterized in terms of linear and nonlinear functions of time over 31 fiscal quarters. Aim 2 sought to examine the impact of system-level policy regulatory activity on the volume of EBP implementation across agencies. Data extracted from site visits in 2014–2016 indexed whether agencies received quality improvement plans indicating the need for corrective actions in the implementation of PEI services. Aim 3 sought to examine the extent to which the volume of EBP implementation was related to overall availability of state funding for public MHS. Data on the availability of state mental health funding were indexed by yearly Medicaid mental health expenditures not tied to PEI implementation requirements. This study extends literature by examining these outer-context determinants of the trajectories of service volume associated with a reimbursement-driven EBP implementation strategy over time.

## Method

### Procedure

Data were part of the Knowledge Exchange on Evidence-Based Practice Sustainment (4KEEPS) Project examining predictors of sustainment of multiple EBPs in a system-driven implementation in children’s MHS [30]. The current study utilized the following data sources: (1) administrative claims billed to PEI funds, (2) regulatory activity data from agency site visits conducted by LACDMH’s PEI Implementation Unit, and (3) California state mental health expenditures. Study procedures were approved by multiple institutional review boards and the Human Subjects Research Committee at LACDMH.

#### Administrative claims data

All claims submitted between April 1, 2010 and December 31, 2017 to PEI funds for services provided to youth 25 years of age or younger were extracted from LACDMH. A total of 6,914,533 claims were available, but only psychotherapy claims (72% of claims) were used for analyses (i.e., claims for other services such as medication management were excluded). As part of the PEI initiative, therapists billed for reimbursement for delivering approved EBPs with a specialized claiming code for each EBP. The primary outcome was the use of the reimbursement implementation strategy, indexed by EBP claims volume. Total psychotherapy claims per agency per fiscal quarter (FQ) was calculated to indicate total volume of EBP claims per FQ at the agency level.

#### System-level regulatory activity

LACDMH provided data from the second round of technical site visits conducted between 2014 and 2016. The purpose of these visits was to assess implementation milestones and provide feedback and assistance with complying with PEI regulations. When indicated, LACDMH provided a Quality Improvement Plan (QIP) to an agency post visit, which included recommendations to ensure that PEI claims were only submitted when services provided and clients served were aligned with regulations [15]. Agencies then responded to LACDMH with corrective action plans. In total, 133 site visits were conducted by LACDMH, and 64 (48.1%) sites/agencies received a QIP requiring a response. LACDMH provided data on the name of the agencies, dates of site visits, the issuance of QIPs, and dates of when formal agency responses to QIPs were due to LACDMH. Site visit data were matched to claims data based on agency identifiers. Because each QIP had a corresponding date, a variable indexing the presence or absence of QIP/regulatory compliance was coded for each agency per FQ, where 0 was coded for all FQ prior to the QIP date, and 1 was coded for all FQ after the QIP date. Agencies that did not receive a QIP were coded 0 across all FQs.

#### State-level MHS funding

Data on California’s state Medi-Cal (the state Medicaid program) mental health expenditures were extracted from the California Department of Health Care Services (www.dhcs.ca.gov/dataandstats/). Total state expenditures (i.e., state general funds and other state funds, which can include MHSA funds) for MHS were extracted for fiscal years (FY) 2009–2010 to FY 2017–2018. Federal Medicaid funds were not included. Expenditures were used as an indicator of available general state Medicaid funding for MHS as a marker of the outer context fiscal climate for each FY of the study.

### Data analytic plan

To describe the trajectory of EBP claims volume (Aim 1), a three-level multilevel regression model was conducted using STATA SE 15.1 to predict EBP claims volume per quarter. The three levels were specified with FQ at level 1, FY at level 2, and agency at level 3 (*n* = 344). A null model indicated significant variance in EBP claims volume at level 2 (FY; *ICC* = .80) and level 3 (agency; *ICC* = .62). Two nested time variables were specified to allow for the treatment of time in a flexible manner [31] and to account for the dependency between outcomes at the FQ level in a given year. The volume of psychotherapy claims per agency per FQ was modeled including linear, quadratic, and cubic functions of time, and state Medicaid MHS expenditures over time were examined using descriptive statistics. To examine the relationship between county-level regulatory activity (Aim 2) and Medicaid MHS expenditures (Aim 3) on EBP claims volume, regulatory activity, and FY state-level mental health expenditures were examined as predictors of EBP claims volume within FQs.

## Results

### Aim 1: describe EBP claims volume and state Medicaid mental health expenditures across time

**Fig. 2:**
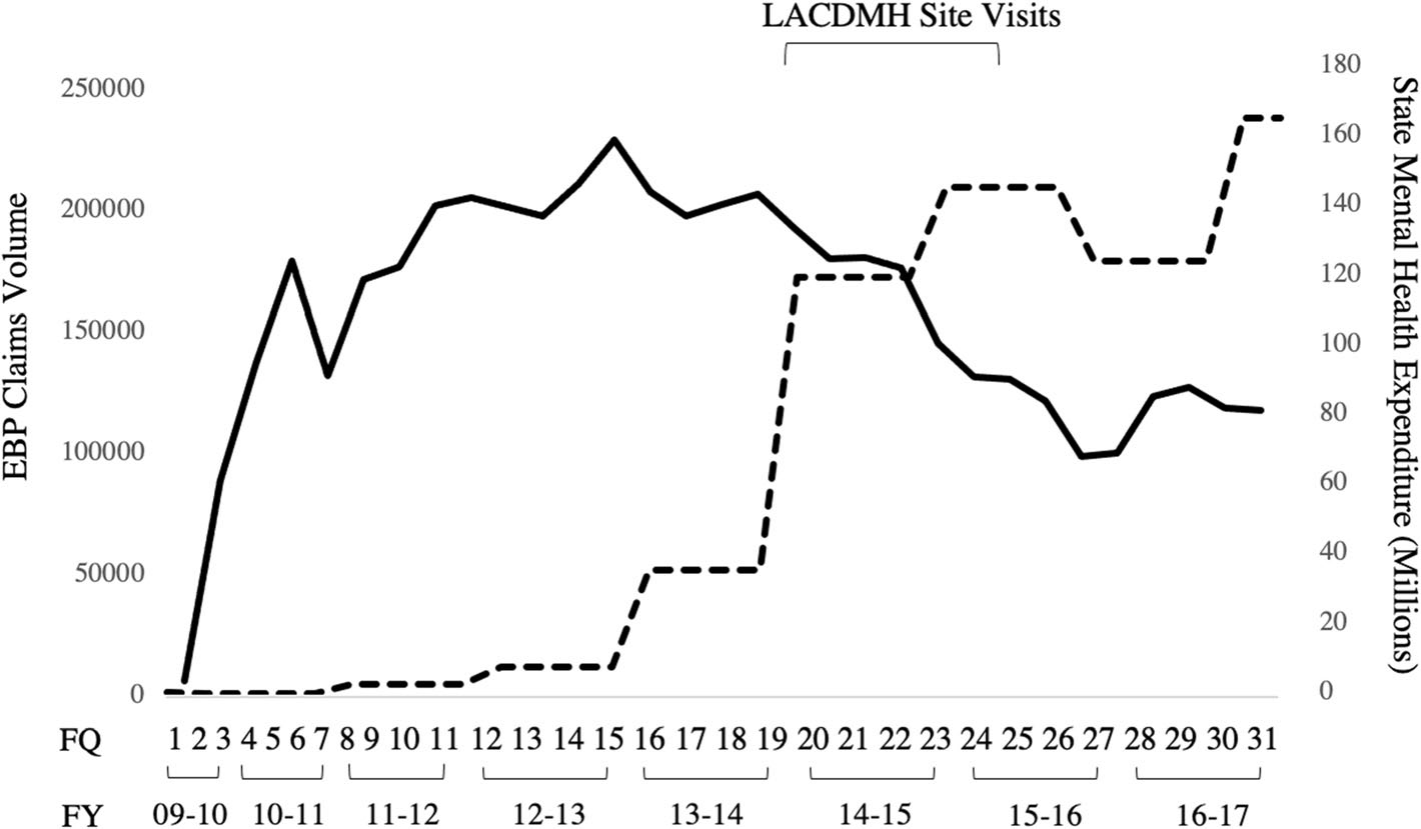
Total claims volume and state mental health expenditure from April 2010 to December 2017. Note: Solid line represents EBPS claims volume, and dotted line represents state mental health expenditure. Site visits occurred between FQs 19–25

Data consisted of 4,912,110 psychotherapy claims submitted for PEI reimbursement between April 1, 2010 and December 31, 2017 (31 FQs). In total, 158,231 children were served by 12,240 therapists across 366 agencies. A three-level regression model was conducted to predict EBP claims volume across 31 FQs with linear, quadratic, and cubic functions of time. Results indicated that claims volume had a significant curvilinear relationship across time. Specifically, the linear component (*B* = 158.18, *p* < .001), the quadratic component (*B* = − 10.50, *p* < .001) and the cubic component (*B* = .19, *p* < .001) were all significant. The volume of claims significantly increased between FQ1 (April 2010) and FQ12 (March 2013), significantly decreased between FQ13 (April 2013) and FQ28 (March 2017), and significantly increased slightly between FQ29 (April 2017) and FQ31 (December 2017) (See Fig. 2).

Mental health expenditure significantly increased across time, from $31,510 in 2010 to $165,197,870 in 2017 (Fig. 2). Thus, more funds became available for MHS expenditures in the state across the study period.

### Aim 2: examine the relationship between system-level regulatory activity and EBP claims volume

System-level regulatory activity was added as a predictor to the multilevel regression model with linear, quadric, and cubic functions of time. Regulatory compliance activity directed toward an agency following the site visit was inversely related to EBP claims volume in the ensuring FQs (*B* = − 99.55, *p* <.001).

### Aim 3: examine the relationship between state Medicaid mental health expenditures and EBP claims volume

State Medicaid mental health expenditure each year was simultaneously added as a predictor to the previous multilevel regression model. State Medicaid mental health expenditures in a given year were inversely related to the volume of EBP delivery within corresponding FQs (*B* = − .87, *p* = .02). As state mental health funding increased across the years, fewer EBP claims were made to the PEI funding stream (Fig. 2).

## Discussion

This study occurred in the USA where healthcare is commonly provided on a fee-for-service model [27]. We sought to examine how changes in outer-context drivers, namely county-level regulation and state-level MHS funding landscape, influenced a reimbursement implementation strategy to increase the delivery of child EBPs. Past literature focused on inner-context drivers of EBP implementation [7]. This study extends the literature by examining how outer-context state mental health funding and county policy enactment impacted EBP delivery driven by a local fiscal implementation strategy. We first sought to describe EBP implementation over time within a county-wide reimbursement-driven strategy to implement EBPs. Results indicated a curvilinear pattern of EBP claims volume over 8 years, with rapid increase in claims volume during the initial scale-up phase, followed by a steady decrease in claims volume in the sustainment phase. This pattern is perhaps unsurprising and mirrors findings of partial sustainment being the most commonly observed outcome among reviewed studies [32]. The drop-off from initial implementation to sustainment may be attributed to decrease in implementation supports or changes in priorities or resource availability over time [32]. Importantly, in the current study, the downward trajectory leveled off, and claims volume increased slightly in the final year of the study period. Thus, although EBP volume did not remain at peak levels, it nonetheless remained above pre-implementation level 8 years later.

The second aim was to examine how county-level regulatory activity indexed by whether an agency received a QIP to comply with implementation guidelines may impact the use of the fiscal implementation strategy. Results indicated nearly half of agencies received a directive to adjust their PEI service provision, which significantly contributed to decreased subsequent EBP claims volume. Site visits conducted by LACDMH between 2014 and 2016 emphasized compliance with guidelines on PEI target population eligibility. PEI services are intended for a prevention and early intervention population where symptomology reflects early-stage illness with clients who can benefit from time-limited delivery of EBPs. Instances where services provided by agencies were out-of-compliance–triggered QIPs, such as developing steps to modify intake procedures and conducting regular audits to determine PEI eligibility. Because the site visits were focused on regulatory compliance, it was understandable that this resulted in the decrease in EBP claims volume and illustrates the effects of a system policy regulatory driver on the use of an implementation strategy at the local level.

The third aim was to examine how state mental health funding, indexed by state mental health expenditures, was associated with the sustainment of the county policy-driven fiscal implementation strategy. Results revealed that EBP claims volume decreased as state mental health funding availability increased, after accounting for local regulatory activity. The finding that EBP implementation ebbed as resources were more available may sound counterintuitive. However, this finding suggests that EBP implementation policy initiatives translated as a fee-for-service reimbursement strategy is sensitive to outer-context fluctuations in unrestricted funding availability. As alternative funding resources became more plentiful, the use of the reimbursement-driven implementation strategy decreased. Our finding has implications for systems employing a reimbursement-driven strategy for EBP implementation. During times of scarce resources, such as the state budget crisis which prevailed the start of the PEI initiative, employing a reimbursement-driven implementation strategy to roll out EBPs may be particularly potent. Thousands of therapists became trained in and delivered EBPs in order to have services reimbursed [33]. However, as the state economy improved, agencies may opt to deliver care with fewer restrictions that did not require EBP delivery. This is particularly likely when the reimbursement rate was equivalent regardless of EBP delivery and when there are significant ongoing costs of EBP implementation associated with provider training, outcome monitoring, and documentation [34].

Within the context of a multi-payer system for healthcare, there is increasing exploration of alternative financing strategies to fee-for-service for incentivizing evidence-based care. One strategy is to provide enhanced reimbursement rates for EBP delivery (e.g., Community Care in Pittsburg). Although enhanced rates are often perceived as effective, they are not commonly employed [35]. Stewart et al. [35] found that only 11% of states in the USA use enhanced reimbursement rates to promote the use of EBPs. An alternative approach is performance-based contracting, where providers are paid when they meet pre-specified performance targets, such as metrics related to child outcomes or service efficiency (e.g., % retention in treatment, timeliness of service) (e.g., Washington State Department of Social and Health Services for child welfare services) [36]. Research on the impact of this financing scheme on increasing the use of EBPs and child outcomes in Washington State are ongoing [37]. A case study of performance-based contract to procure EBP in child welfare services in California found positive and negative perceptions among administrators. Positive perceptions included greater control of decision-making over service provision within agencies and agencies becoming more outcome-oriented as a result of changes in reporting requirements [38].

Another fiscal strategy is the pay-for-success (PFS) mechanism, where private investors fund the initial implementation of an EBP and subsequently receive a return from a government payer if the program results in significant improvements in outcomes based on an independent evaluation [39]. Segal et al. [39] reviewed eleven existing programs that spans service sectors in the USA financed by PFS. Of the three programs that reached the payout decision point, two programs demonstrated success and received payouts [39]. Recently, Dopp et al. [40] described the applicability of PFS financing for implementing Multisystemic Therapy, an EBP for antisocial behavior for youth [41]. While many fiscal implementation strategies have been identified, there still remains limited understanding of their effectiveness for achieving long-term sustainment of EBPs.

### Limitations

Several limitations should be noted when interpreting findings of the study. EBP delivery was indexed by claims, which precludes conclusions about the quality, extent, or fidelity of EBP delivery. Claims data was limited to a single funding source (i.e., PEI), and agencies received reimbursement from a variety of other funding sources during the study period. It is possible and likely that therapists continue to use EBPs when claiming for reimbursement from other funding sources not captured in our data. Thus, current findings of EBP volume likely reflect an underestimation of actual EBP delivery, though findings still illustrate agency engagement with the fiscal implementation reimbursement strategy. Furthermore, PEI funds catalyzed the introduction of EBPs in LACDMH, but the delivery of EBPs has likely expanded throughout the system of care since PEI was introduced. Indeed, Kim et al. [42] found evidence of ‘generalizing adaptations’ in the LACDMH context where therapists applied EBPs in alternate settings, to alternative problem focus, and with alternate individuals than typically intended, suggesting flexible use and some generalization of EBP implementation. Another important indicator of EBP implementation and sustainment is reach. The current study did not have available data regarding the number of youth *eligible* to receive EBP to be able to adequately assess reach. The study occurred in the USA where healthcare is financed by a combination of private and public insurers. A reimbursement-based implementation strategy may have less influence in other contexts where healthcare is structured and financed differently, such as a single payer system, socialized medicine, or systems with different compositions of health spending from government, private, out-of-pocket, and development assistance/philanthropy. The specific reimbursement-based strategy described in LA county may not be representative of other counties or states in the USA or in other countries and may have most relevance to health care systems that have elements of fee-for-service financing structures. With the introduction of PEI, LACDMH and contracted agencies implemented a cascade of organizational and structural changes to support workforce development and implementation of EBPs aside from the reimbursement mechanism, which also contributed to sustained EBP delivery [33, 43]. Data on mental health expenditure only included state Medicaid funding for MHS. Inclusion of a comprehensive portfolio of funding sources for public mental health may have yielded different results. The current study only considered EBPs delivered in children’s MHS. Previous studies suggested the possibility that outer-context determinants may differ for adult-versus-child services [12]. Finally, the present study examined the outcomes of a county policy implementing a fiscal implementation strategy as it interacted with the state MHS funding context, further study is needed to determine if this type of cross-level interaction would hold in other county and local systems.

### Implications

Findings have implications for policymakers and system leaders when mobilizing fiscal implementation strategies for promoting EBP use and further highlight the importance of considering outer-context drivers. When choosing to employ a reimbursement-driven implementation strategy, it may be important to consider additional incentives (e.g., enhanced rates, cost-based rates, or other structures) to facilitate EBP sustainment given that this strategy is sensitive to fiscal health fluctuations, resource availability, and system level policies. The principle of the relative potency of employing a fiscal implementation strategy to implement evidence-based care also applies to healthcare systems with different financing structures.

## Data Availability

Data analyzed in this study are owned by the Los Angeles Department of Mental Health (LACDMH), and as such, are not publicly available. Data may be available from LACDMH upon reasonable request and with permission.
